# Supplementary material for: Mucosal Responses to Zika Virus Infection in Cynomolgus Macaques
Source: Pathogens. 2022 Sep 12;11(9):1033. doi: 10.3390/pathogens11091033 (PMC9503824; doi:10.3390/pathogens11091033)
Supplement: Supplementary file 1 [file pathogens-11-01033-s001.zip › Figure S1.pdf]

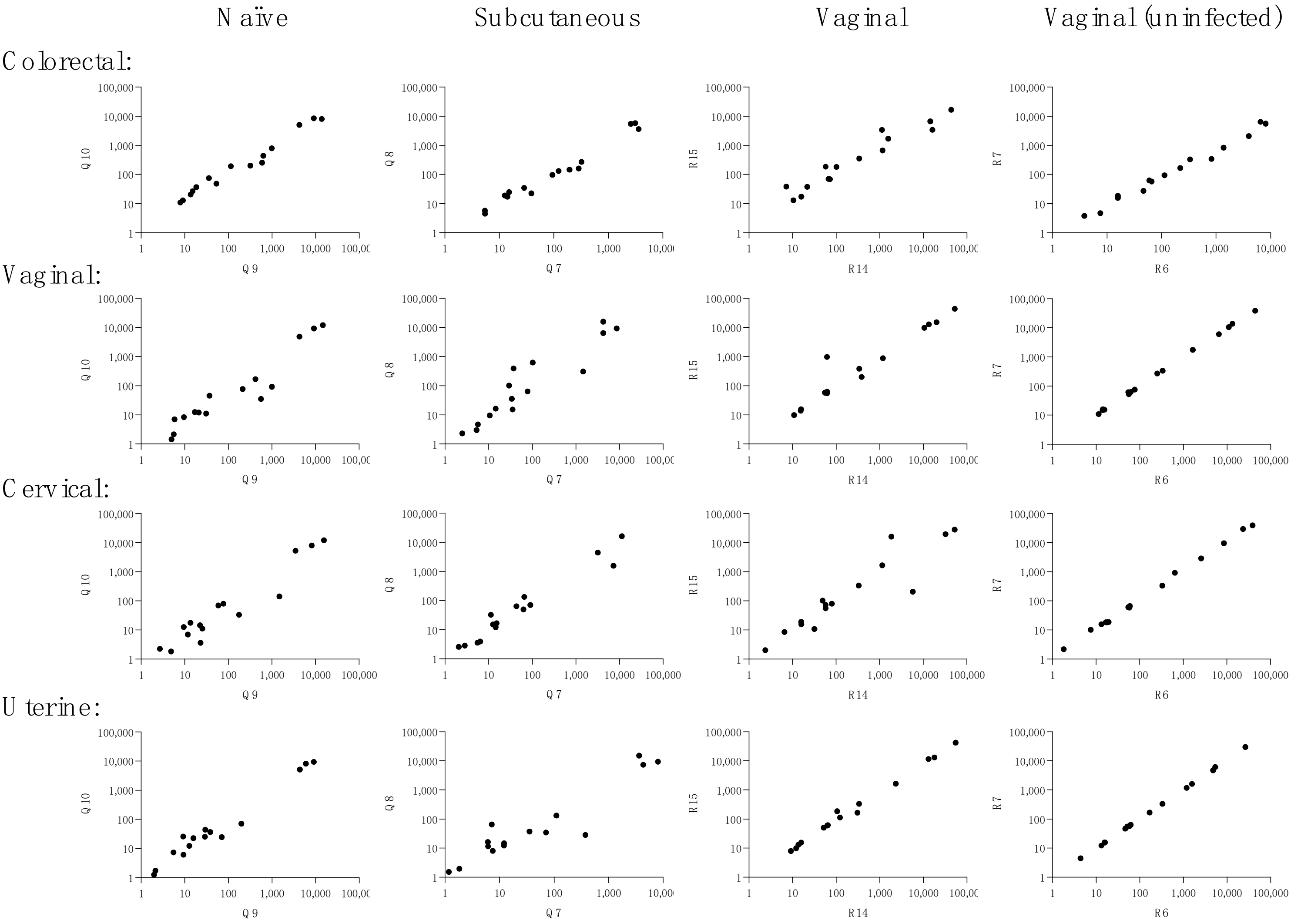

Figure S1. Interindividual variability of proteomic responses to ZIKV challenge per study group. Spearman correlation was performed between cytokine concentrations from each macaque within each group (naïve: Q10 and Q9; subcutaneous ZIKV challenge: Q8 and Q7; vaginal ZIKV challenge: R15 and R14; uninfected following vaginal ZIKV challenge: R7 and R6) measured in culture supernatants from colorectal, vaginal, cervical and uterine tissue explants.
