# Supplementary material for: Mucosal Responses to Zika Virus Infection in Cynomolgus Macaques
Source: Pathogens. 2022 Sep 12;11(9):1033. doi: 10.3390/pathogens11091033 (PMC9503824; doi:10.3390/pathogens11091033)
Supplement: Supplementary file 1 [file pathogens-11-01033-s001.zip › Figure S2.pdf]

(a)

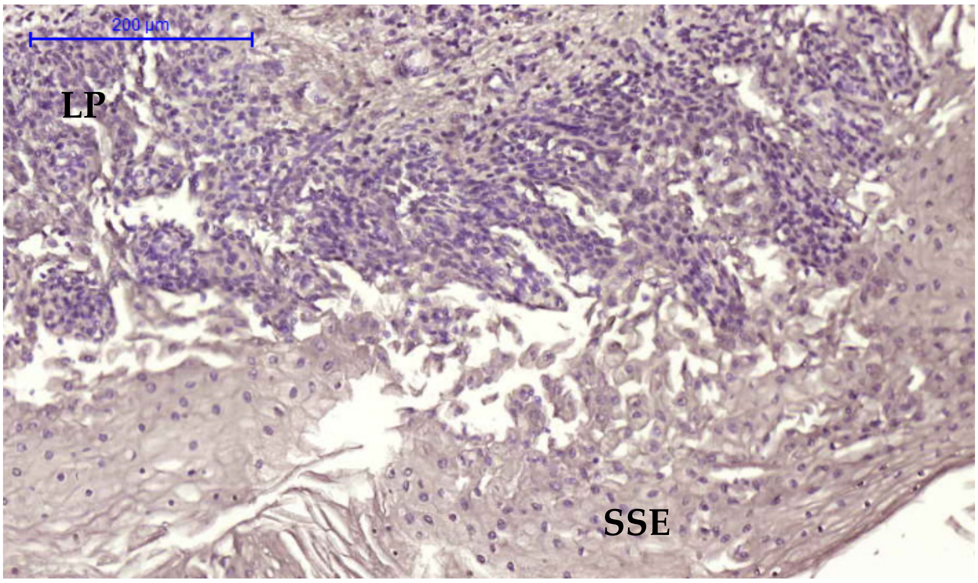

(b)

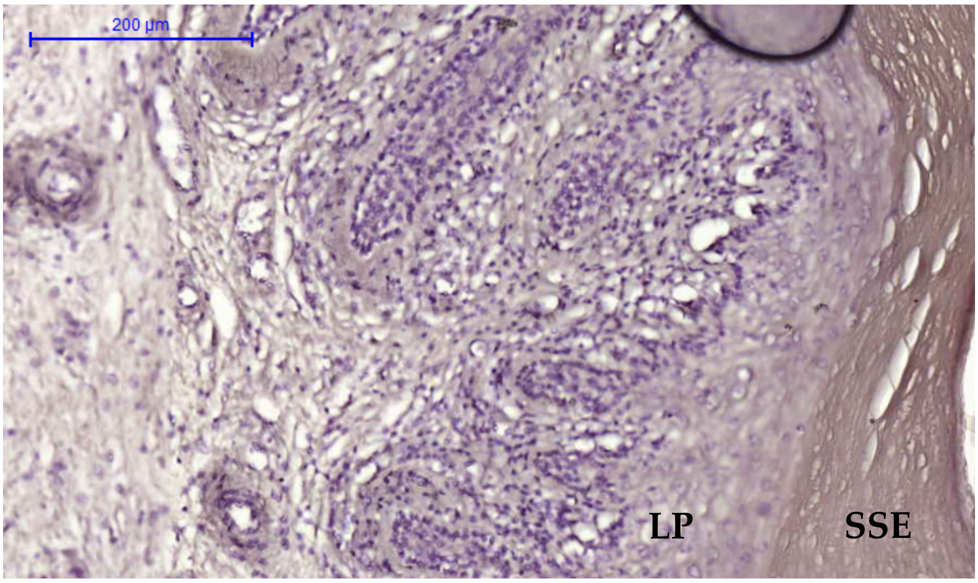

Figure S2. RNAscope In Situ Hybridization negative controls. Vaginal explants were cultured for 1 day either (**a**) unchallenged or (**b**) exposed to 10^5 pfu of ZIKV PRABC59 for 2 h. Representative images of sections processed for RNAscope analysis in (**a**) the presence or (**b**) absence of probe but with nuclei stained with haematoxylin (blue). Blue bar = 200 µm. SSE: stratified squamous epithelium; LP: lamina propria**.**
