# Supplementary material for: Mucosal Responses to Zika Virus Infection in Cynomolgus Macaques
Source: Pathogens. 2022 Sep 12;11(9):1033. doi: 10.3390/pathogens11091033 (PMC9503824; doi:10.3390/pathogens11091033)
Supplement: Supplementary file 1 [file pathogens-11-01033-s001.zip › Supplementary Table S1.pdf]

**Table S1.** Mucosal cytokine responses to *in vivo* ZIKV exposure [1].

Tissue: Colorectal

| ZIKV challenge | Cytokine | Virus vs. Ctrl FC | *P* value |
|---|---|---|---|
| Subcutaneous | GM-CSF | 0.399 | 0.27370 |
| | IFN-γ | 0.567 | 0.35040 |
| | IL-2 | 0.920 | 0.79758 |
| | IL-15 | 0.746 | 0.47389 |
| | IL-17 | 0.592 | 0.12627 |
| | IL-1ra | 0.485 | 0.14821 |
| | IL-1β | 0.602 | 0.14109 |
| | IL-5 | 0.717 | 0.53290 |
| | IL-6 | 0.458 | 0.08282 |
| | IL-8 | 0.329 | 0.12518 |
| | MCP-1 | 0.958 | 0.90168 |
| | MIP-1β | 0.453 | 0.09285 |
| | VEGF-A | 0.250 | 0.02942 |
| | IL-4 | 0.623 | 0.28134 |
| | IL-10 | 0.551 | 0.13676 |
| Vaginal | GM-CSF | 2.153 | 0.10653 |
| | IFN-γ | 2.552 | 0.07918 |
| | IL-2 | 4.105 | 0.00020 |
| | IL-15 | 0.561 | 0.07125 |
| | IL-17 | 3.174 | 0.04847 |
| | IL-1ra | 8.672 | 0.11380 |
| | IL-1β | 2.394 | 0.27339 |
| | IL-5 | 0.599 | 0.12514 |
| | IL-6 | 3.416 | 0.14327 |
| | IL-8 | 0.893 | 0.85175 |
| | MCP-1 | 2.268 | 0.15493 |
| | MIP-1β | 31.518 | 0.00000 |
| | VEGF-A | 1.828 | 0.00440 |
| | IL-4 | 0.447 | 0.02512 |
| | IL-10 | 0.042 | 0.00105 |
| Vaginal (uninfected) | GM-CSF | 0.455 | 0.30850 |
| | IFN-γ | 1.112 | 0.78718 |
| | IL-2 | 2.164 | 0.18709 |
| | IL-15 | 0.292 | 0.13345 |
| | IL-17 | 1.826 | 0.06051 |
| | IL-1ra | 4.234 | 0.09048 |
| | IL-1β | 2.033 | 0.03494 |
| | IL-5 | 0.567 | 0.32682 |
| | IL-6 | 0.778 | 0.26842 |
| | IL-8 | 0.277 | 0.12048 |
| | MCP-1 | 1.393 | 0.04997 |
| | MIP-1β | 30.271 | 0.00007 |
| | VEGF-A | 0.654 | 0.35544 |
| | IL-4 | 0.398 | 0.14498 |
| | IL-10 | 0.007 | 0.03107 |

Tissue: Vaginal

| ZIKV challenge | Cytokine | Virus vs. Ctrl FC | *P* value |
|---|---|---|---|
| Subcutaneous | GM-CSF | 1.482 | 0.74853 |
| | IFN-γ | 1.121 | 0.30044 |
| | IL-2 | 1.044 | 0.81526 |
| | IL-15 | 1.538 | 0.50217 |
| | IL-17 | 1.065 | 0.91412 |
| | IL-1ra | 1.640 | 0.68273 |
| | IL-1β | 3.377 | 0.05465 |
| | IL-5 | 0.807 | 0.25777 |
| | IL-6 | 0.970 | 0.48965 |
| | IL-8 | 0.755 | 0.63778 |
| | MCP-1 | 1.167 | 0.57235 |
| | MIP-1β | 0.750 | 0.69413 |
| | VEGF-A | 0.220 | 0.47318 |
| | IL-4 | 0.845 | 0.28484 |
| | IL-10 | 1.249 | 0.82541 |
| Vaginal | GM-CSF | 2.009 | 0.18285 |
| | IFN-γ | 6.512 | 0.00007 |
| | IL-2 | 4.127 | 0.00043 |
| | IL-15 | 0.626 | 0.09253 |
| | IL-17 | 3.784 | 0.00091 |
| | IL-1ra | 18.721 | 0.00007 |
| | IL-1β | 24.585 | 0.27703 |
| | IL-5 | 2.422 | 0.00002 |
| | IL-6 | 5.206 | 0.00078 |
| | IL-8 | 1.320 | 0.18684 |
| | MCP-1 | 2.861 | 0.00003 |
| | MIP-1β | 112.680 | 0.00005 |
| | VEGF-A | 3.485 | 0.02610 |
| | IL-4 | 1.510 | 0.00146 |
| | IL-10 | 0.193 | 0.03617 |
| Vaginal (uninfected) | GM-CSF | 1.794 | 0.23480 |
| | IFN-γ | 6.473 | 0.00514 |
| | IL-2 | 3.694 | 0.00533 |
| | IL-15 | 0.676 | 0.35121 |
| | IL-17 | 3.728 | 0.02685 |
| | IL-1ra | 11.619 | 0.00783 |
| | IL-1β | 3.600 | 0.03136 |
| | IL-5 | 2.406 | 0.00473 |
| | IL-6 | 4.501 | 0.00876 |
| | IL-8 | 0.806 | 0.19529 |
| | MCP-1 | 2.956 | 0.00164 |
| | MIP-1β | 104.719 | 0.00003 |
| | VEGF-A | 5.728 | 0.03413 |
| | IL-4 | 1.515 | 0.03931 |
| | IL-10 | 0.051 | 0.15515 |

Tissue: Cervical

| ZIKV challenge | Cytokine | Virus vs. Ctrl FC | *P* value |
|---|---|---|---|
| Subcutaneous | GM-CSF | 0.340 | 0.46252 |
| | IFN-γ | 1.253 | 0.30113 |
| | IL-2 | 0.731 | 0.56755 |
| | IL-15 | 1.019 | 0.90289 |
| | IL-17 | 1.159 | 0.22049 |
| | IL-1ra | 0.123 | 0.40006 |
| | IL-1β | 0.550 | 0.25703 |
| | IL-5 | 1.186 | 0.79136 |
| | IL-6 | 0.550 | 0.33238 |
| | IL-8 | 0.995 | 0.98248 |
| | MCP-1 | 0.875 | 0.66782 |
| | MIP-1β | 0.691 | 0.56985 |
| | VEGF-A | 0.514 | 0.55342 |
| | IL-4 | 0.872 | 0.42893 |
| | IL-10 | 1.029 | 0.84283 |
| Vaginal | GM-CSF | 1.592 | 0.50718 |
| | IFN-γ | 5.878 | 0.00318 |
| | IL-2 | 4.363 | 0.00018 |
| | IL-15 | 0.481 | 0.01078 |
| | IL-17 | 7.056 | 0.00068 |
| | IL-1ra | 11.055 | 0.25676 |
| | IL-1β | 8.091 | 0.04854 |
| | IL-5 | 0.851 | 0.32088 |
| | IL-6 | 4.981 | 0.03989 |
| | IL-8 | 1.872 | 0.10807 |
| | MCP-1 | 0.681 | 0.60266 |
| | MIP-1β | 99.358 | 0.00000 |
| | VEGF-A | 13.455 | 0.00581 |
| | IL-4 | 0.878 | 0.07949 |
| | IL-10 | 0.028 | 0.00000 |
| Vaginal (uninfected) | GM-CSF | 1.311 | 0.71198 |
| | IFN-γ | 5.213 | 0.00469 |
| | IL-2 | 3.431 | 0.03065 |
| | IL-15 | 0.564 | 0.10973 |
| | IL-17 | 5.894 | 0.01212 |
| | IL-1ra | 0.963 | 0.96867 |
| | IL-1β | 2.029 | 0.05683 |
| | IL-5 | 0.984 | 0.94621 |
| | IL-6 | 4.819 | 0.00002 |
| | IL-8 | 1.904 | 0.06719 |
| | MCP-1 | 2.080 | 0.04487 |
| | MIP-1β | 99.018 | 0.00004 |
| | VEGF-A | 26.030 | 0.00438 |
| | IL-4 | 0.908 | 0.37807 |
| | IL-10 | 0.025 | 0.00025 |

Tissue: Uterine

| ZIKV challenge | Cytokine | Virus vs. Ctrl FC | *P* value |
|---|---|---|---|
| Subcutaneous | GM-CSF | 0.987 | 0.98917 |
| | IFN-γ | 1.215 | 0.29025 |
| | IL-2 | 1.073 | 0.56398 |
| | IL-15 | 0.635 | 0.17422 |
| | IL-17 | 0.989 | 0.92840 |
| | IL-1ra | 1.109 | 0.87560 |
| | IL-1β | 1.143 | 0.75709 |
| | IL-5 | 0.644 | 0.58610 |
| | IL-6 | 0.939 | 0.47107 |
| | IL-8 | 1.322 | 0.73311 |
| | MCP-1 | 1.222 | 0.55883 |
| | MIP-1β | 0.838 | 0.58586 |
| | VEGF-A | 7.464 | 0.42031 |
| | IL-4 | 0.967 | 0.39898 |
| | IL-10 | 0.893 | 0.84500 |
| Vaginal | GM-CSF | 6.432 | 0.03138 |
| | IFN-γ | 7.991 | 0.00000 |
| | IL-2 | 5.043 | 0.00001 |
| | IL-15 | 0.447 | 0.00789 |
| | IL-17 | 6.984 | 0.00006 |
| | IL-1ra | 41.707 | 0.00327 |
| | IL-1β | 15.160 | 0.00002 |
| | IL-5 | 0.901 | 0.74880 |
| | IL-6 | 5.223 | 0.01186 |
| | IL-8 | 2.180 | 0.03982 |
| | MCP-1 | 2.586 | 0.00095 |
| | MIP-1β | 206.807 | 0.00000 |
| | VEGF-A | 5.357 | 0.02731 |
| | IL-4 | 1.627 | 0.00011 |
| | IL-10 | 0.081 | 0.03437 |
| Vaginal (uninfected) | GM-CSF | 4.555 | 0.00312 |
| | IFN-γ | 8.318 | 0.00127 |
| | IL-2 | 3.721 | 0.00004 |
| | IL-15 | 0.826 | 0.42756 |
| | IL-17 | 6.641 | 0.00197 |
| | IL-1ra | 24.874 | 0.00044 |
| | IL-1β | 8.119 | 0.00099 |
| | IL-5 | 0.897 | 0.84656 |
| | IL-6 | 3.033 | 0.00788 |
| | IL-8 | 0.672 | 0.16355 |
| | MCP-1 | 1.205 | 0.20324 |
| | MIP-1β | 205.749 | 0.00001 |
| | VEGF-A | 58.861 | 0.00012 |
| | IL-4 | 1.509 | 0.00213 |
| | IL-10 | 0.033 | 0.17892 |

[1]Significantly modulated genes as determined by unpaired *t* test with a Benjemini-Hochberg multiple testing correction ($P < 0.05$)

FC: Fold change
