# Supplementary material for: Mucosal Responses to Zika Virus Infection in Cynomolgus Macaques
Source: Pathogens. 2022 Sep 12;11(9):1033. doi: 10.3390/pathogens11091033 (PMC9503824; doi:10.3390/pathogens11091033)
Supplement: Supplementary file 1 [file pathogens-11-01033-s001.zip › Supplementary Table S2.pdf]

Table S2. Interindividual variability analysis within study groups[1].

Tissue	ZIKV challenge	r_S	*P* value
Colorectal	Naïve	0.9929	<0.0001
	Subcutaneous	0.9714	<0.0001
	Vaginal	0.9393	<0.0001
	Vaginal (uninfected)	0.9929	<0.0001
Vaginal	Naïve	0.9571	<0.0001
	Subcutaneous	0.9393	<0.0001
	Vaginal	0.9107	<0.0001
	Vaginal (uninfected)	0.9929	<0.0001
Cervical	Naïve	0.9071	<0.0001
	Subcutaneous	0.9643	<0.0001
	Vaginal	0.9429	<0.0001
	Vaginal (uninfected)	0.9929	<0.0001
Uterine	Naïve	0.9071	<0.0001
	Subcutaneous	0.8321	0.0002
	Vaginal	0.9893	<0.0001
	Vaginal (uninfected)	0.9893	<0.0001

[1]Spearman correlations between cytokine levels from macaques of each group; r_S: Spearman correlation coefficient; P value determined using a two-tailed unpaired Student's *t* test.
