# Supplementary material for: Mucosal Responses to Zika Virus Infection in Cynomolgus Macaques
Source: Pathogens. 2022 Sep 12;11(9):1033. doi: 10.3390/pathogens11091033 (PMC9503824; doi:10.3390/pathogens11091033)
Supplement: Supplementary file 1 [file pathogens-11-01033-s001.zip › Supplementary Table S3.pdf]

Table S3. Mucosal cytokine responses to *ex vivo* ZIKV exposure [1].

Tissue: Colorectal

ZIKV challenge	Cytokine	Virus vs. Ctrl FC	*P* value
Ex vivo	GM-CSF	0.820	0.76416
	IFN-γ	0.837	0.71531
	IL-2	0.982	0.94168
	IL-15	0.876	0.72006
	IL-17	0.804	0.41496
	IL-1ra	0.643	0.27233
	IL-1β	0.608	0.14489
	IL-5	1.324	0.60781
	IL-6	0.756	0.04693
	IL-8	0.603	0.32395
	MCP-1	0.974	0.89240
	MIP-1β	0.824	0.56254
	VEGF-A	0.414	0.03758
	IL-4	0.961	0.89567
	IL-10	0.760	0.40044

Tissue: Vaginal

ZIKV challenge	Cytokine	Virus vs. Ctrl FC	*P* value
Ex vivo	GM-CSF	1.170	0.75227
	IFN-γ	2.546	0.00632
	IL-2	1.692	0.04996
	IL-15	0.830	0.59347
	IL-17	1.649	0.28001
	IL-1ra	3.138	0.12412
	IL-1β	2.737	0.06652
	IL-5	1.205	0.17038
	IL-6	1.393	0.00870
	IL-8	1.004	0.98059
	MCP-1	1.550	0.02296
	MIP-1β	4.227	0.02777
	VEGF-A	1.715	0.50341
	IL-4	1.041	0.73553
	IL-10	0.479	0.34512

Tissue: Cervical

ZIKV challenge	Cytokine	Virus vs. Ctrl FC	*P* value
Ex vivo	GM-CSF	0.464	0.53845
	IFN-γ	1.678	0.07817
	IL-2	0.843	0.75787
	IL-15	1.224	0.64206
	IL-17	1.285	0.11380
	IL-1ra	0.753	0.82172
	IL-1β	0.814	0.55495
	IL-5	1.255	0.70250
	IL-6	0.676	0.58385
	IL-8	0.956	0.81782
	MCP-1	0.644	0.60743
	MIP-1β	1.372	0.65542
	VEGF-A	3.412	0.51916
	IL-4	1.158	0.64969
	IL-10	1.245	0.09749

Tissue: Uterine

ZIKV challenge	Cytokine	Virus vs. Ctrl FC	*P* value
Ex vivo	GM-CSF	0.955	0.89270
	IFN-γ	1.036	0.88357
	IL-2	1.059	0.51401
	IL-15	1.455	0.31415
	IL-17	0.924	0.71360
	IL-1ra	1.392	0.50365
	IL-1β	1.082	0.75657
	IL-5	1.069	0.94495
	IL-6	0.970	0.26880
	IL-8	1.011	0.98450
	MCP-1	0.995	0.96617
	MIP-1β	0.879	0.68666
	VEGF-A	0.916	0.38754
	IL-4	0.918	0.12716
	IL-10	0.583	0.47974

[1]Significantly modulated genes as determined by unpaired *t* test with a Benjemini-Hochberg multiple testing correction ($P < 0.05$)

FC: Fold change
